# Flavonoids as Structural Probes Reveal Conformationally Dependent Ligand Recognition in the SARS-CoV-2 JN.1 Spike Protein

**DOI:** 10.3390/ijms27156624

**Published:** 2026-07-24

**Authors:** Susana R. Castro-Jiménez, Armando Mejía, Carlos Cabello, Gabriela Léon-Gutierrez, Cesar Millán-Pacheco

**Affiliations:** 1 Departamento de Biotecnología, Universidad Autónoma Metropolitana-Iztapalapa, Ciudad de México 09340, Mexico; reginacastrojim@gmail.com (S.R.C.-J.); ama@xanum.uam.mx (A.M.); 2 Departamento de Virología e Investigación en Micología, Instituto Nacional de Enfermedades Respiratorias, Ciudad de México 14080, Mexico; carloscg@iner.gob.mx; 3 Inmolecule International Limited, 16 Great Queen Street, Covent Garden, London WC2B 5AH, UK; glg@inmolecule.com; 4 Facultad de Farmacia, Universidad Autónoma del Estado de Morelos, Av. Universidad No. 1001, Col Chamilpa, Cuernavaca 62209, Mexico

**Keywords:** molecular docking, SARS-CoV-2 spike protein, JN.1 subvariant, conformational dynamics, protein–ligand recognition, receptor-binding domain (RBD), structural probes

## Abstract

The SARS-CoV-2 JN.1 subvariant has attracted attention due to the accumulation of mutations in the Spike (S) glycoprotein, particularly within the receptor-binding domain (RBD), contributing to the structural heterogeneity of the Spike protein. However, the extent to which conformational dynamics influence ligand-recognition patterns across functional Spike states remains insufficiently characterized. In this study, we applied a hierarchical computational framework combining homology modeling, molecular dynamics simulations, and molecular docking to determine whether conformational sampling modifies the location, accessibility, and recurrence of ligand-interaction regions in the JN.1 Spike protein. Closed, semi-closed, and open conformations were modeled and subjected to triplicate simulations, including an initial 100 ns phase followed by extended 200 ns simulations. Representative conformations were obtained by trajectory clustering and used for docking analyses. Two flavonoids, hesperitin-7-O-rutinoside (H7R) and flavanone-7-O-glucoside (F7G), were employed as structural probes to assess how ligand interaction patterns vary across conformational states. Comparative analyses revealed that conformational sampling modifies the location, accessibility and spatial distribution of ligand-interaction regions while generating distinct docking poses and recurrent interaction patterns not fully captured by static structural models. These findings highlight the importance of dynamics-informed structural ensembles for docking analyses in flexible viral proteins. Rather than predicting absolute binding affinities, this study provides an exploratory computational framework for evaluating ligand-recognition behavior in structurally dynamic viral systems.

## 1. Introduction

Since its emergence in December 2019, Severe Acute Respiratory Syndrome Coronavirus 2 (SARS-CoV-2) has undergone continuous evolution, leading to the appearance of multiple variants that have significantly influenced the global pandemic. Among them, the Omicron variant (B.1.1.529) has been characterized by high transmissibility and pronounced immune evasion, giving rise to numerous sublineages with distinct virological and structural properties [1,2].

In November 2023, the JN.1 subvariant, a direct descendant of BA.2.86 (Pirola), was designated as a Variant of Interest (VOI) by the World Health Organization (WHO) due to its rapid global dissemination and the accumulation of multiple mutations in the Spike protein [3]. Key substitutions, including L455S and F456L, have been associated with altered ACE2 binding affinity and reduced neutralization by monoclonal and polyclonal antibodies, particularly within the receptor-binding domain (RBD), which plays a central role in viral entry [4,5]. During the first half of 2025, emerging variants such as NB.1.8.1, LP.8.1, XEC, and KP.3.1.1 were reported in different regions worldwide, and phylogenetic analyses indicate that most currently circulating lineages descend from JN.1 [3,6,7].

**Figure 1:**
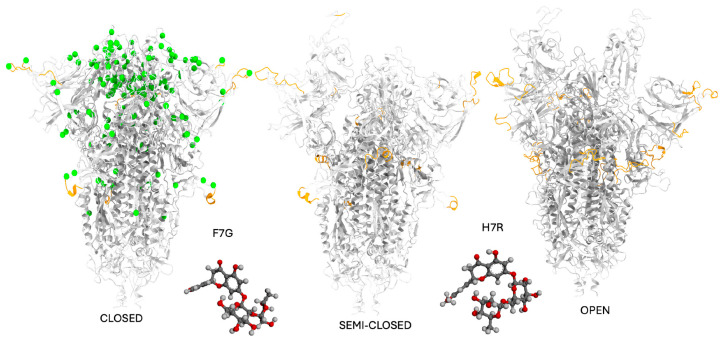
Three-dimensional representation of the functional conformations of the SARS-CoV-2 Spike protein (homology model of the JN.1 subvariant. Green-labeled residues denote characteristic mutations of the JN.1 subvariant compared to the Wuhan strain. Yellow regions correspond to segments not modeled due to a lack of structural coverage in the selected homology modeling template. The bottom inserts display the structures of the flavonoids H7R and F7G. Atoms’ color codes are C (gray), O (red) and H (white).

Rather than acting as a static molecular scaffold, the SARS-CoV-2 Spike protein is a highly dynamic trimeric protein whose biological function is governed by large-scale conformational rearrangements. Spike mediates viral entry through receptor recognition and membrane fusion and is composed of two functional subunits: S1 (which contains the N-terminal domain (NTD) and the RBD and S2, which drives membrane fusion [8,9]. The RBD undergoes reversible transitions between a “down” (closed) conformation, in which (Angiotensin-Converting Enzyme 2) ACE2 binding is sterically hindered, and an “up” (open) conformation that enables receptor engagement [10]. At least three functionally relevant conformational states have been described: closed (all RBDs down), semi-closed (one RBD up), and open (two or more RBDs up) (Figure 1) [11]. These conformations not only modulate ACE2 affinity but also determine the accessibility and geometry of potential ligand-binding sites.

Accumulating evidence indicates that Spike conformational flexibility—particularly within the RBD—is a key determinant of viral infectivity, immune escape, and ligand recognition. Structural interprotomer interactions and mutation-induced rearrangements have been shown to modulate epitope exposure and pocket accessibility [12]. Enhanced-sampling simulations have revealed allosteric communication pathways within the Spike trimer [13], while comparative analyses across Omicron sublineages demonstrate that accumulated mutations progressively reshape the conformational landscape of the protein [14]. These observations challenge docking approaches based on static or minimally relaxed structures.

In this context, JN.1 represents a highly mutated Spike system with pronounced conformational heterogeneity, making it a suitable model to investigate how conformational sampling and structural relaxation influence ligand recognition patterns across different functional states. While homology modeling provides reasonable starting structures, it remains unclear to what extent short molecular dynamics (MD) simulations are sufficient to capture biologically meaningful conformations suitable for interaction analysis.

Natural products, particularly flavonoids, have been widely explored due to their diverse bioactive properties and reported interactions with viral proteins [15]. Previous experimental studies have shown that flavonoid-functionalized materials can interfere with Spike–ACE2 interactions *in vitro*, supporting their potential to interact with the Spike protein [16].

In the present study, flavonoids are not proposed as novel therapeutic candidates but are employed as molecular probes to interrogate the impact of Spike conformational dynamics on ligand recognition. In particular, hesperitin-7-O-rutinoside (H7R) and flavanone-7-O-glucoside (F7G) (Figure 1) were selected based on previous computational and experimental studies suggesting their ability to interact with the Spike protein in the ancestral Wuhan variant [16,17,18,19].

These compounds were chosen due to their well-defined chemical structures and previously reported interaction patterns with viral proteins, making them suitable model ligands to explore how conformational variability influences ligand recognition. Importantly, they are not assumed to directly compete with ACE2 binding. Instead, small molecules such as flavonoids may interact with regions that influence Spike conformational dynamics, modulating transitions between closed, semi-closed, and open states that regulate receptor accessibility.

In this context, H7R and F7G are employed as conformational probes to investigate how conformational sampling and structural context influence ligand-recognition patterns across distinct functional states of the JN.1 Spike protein [13,20,21].

Here, we integrate homology modeling, hierarchical molecular dynamics simulations, and ensemble-based molecular docking to investigate ligand-recognition patterns in the Spike protein of the SARS-CoV-2 JN.1 subvariant.

The central objective of this study was to determine whether conformational sampling modifies the location, accessibility, and recurrence of ligand-interaction regions compared with static structural models. To address this question, ligand interaction patterns obtained from static and dynamically sampled conformations were systematically compared.

As a structural reference framework for these analyses, the Wuhan Spike protein was included due to its extensively characterized structure. Additionally, we sought to characterize the structural dynamics of the closed, semi-closed, and open conformations of the JN.1 Spike protein, assess the reproducibility of docking outcomes through clustering analyses, and compare ligand-recognition patterns obtained from static structures and molecular dynamics-derived conformations. Together, these analyses allowed us to evaluate the impact of conformational sampling on ligand-recognition landscapes in a highly flexible viral protein.

## 2. Results

### 2.1. Structural Modeling of Spike-JN.1

Since no experimentally resolved structure was available for the SARS-CoV-2 JN.1 subvariant at the time of this study, a homology modeling approach was employed using SWISS-MODEL [22,23]. The target sequence (WRK13149.1, NCBI) was aligned with high-resolution crystal structures of the BA.2 subvariant, specifically PDB entries 8D55, 8D56 [24] and 8DM7 [25]. The resulting model spanned residues 14 to 1126 and showed 97.08% identity with the selected templates. GMQE and QSQE are two metrics provided by SWISS-MODEL to estimate the reliability of the model. GMQE value, which combines the expected quality of the alignment and the coverage of the model based on structural templates, a higher value reflects the overall structural confidence of the model. Conversely, QSQE evaluates the expected accuracy in predicting the quaternary structure; a higher value indicates a high reliability in the prediction of inter-subunit interfaces. JN.1 models showed consistent structural quality across conformational states, with GMQE values of 0.71 for both closed and semi-closed conformations and 0.69 for the open state. QSQE values were 0.96 and 0.93 for the closed and semi-closed models, respectively, and 0.94 for the open conformation. Sequence identity remained high across all models, reaching 97.08% for closed and semi-closed states and 96.46% for open conformation. In addition, QMEANDisCo scores were comparable among conformations (0.71 ± 0.05 for closed and semi-closed; 0.74 ± 0.05 for open), supporting the overall reliability of the generated models. Structural analysis confirmed that the models maintained the characteristic trimeric architecture of the Spike protein, with the S1 and S2 subunits organized similarly to previously reported variants. Significant structural differences were observed in flexible loops of the N-terminal domain (NTD), unresolved regions, and segments containing JN.1-specific deletions or insertions (Figure 1).

The three functional trimer conformations—closed, semi-closed, and open—were initially defined based on the orientation of the Receptor-Binding Domain (RBD) (residues 319–541). In the closed state, all three RBDs adopt the down position; in the semi-closed conformation, one RBD is in the up orientation; and in the open conformation, two or more RBDs adopt the up position, increasing ACE2 accessibility. This classification is consistent with previous structural descriptions of the Wuhan variant and with geometric approaches derived from previous molecular dynamics studies [11].

It is important to note that these homology-based models represent reference conformational starting structures and were not used directly for interaction analyses. Instead, they served as initial inputs for molecular dynamics simulations aimed at validating structural stability, refining conformational flexibility, and capturing dynamically representative states, which were subsequently selected through conformational clustering prior to molecular docking studies.

### 2.2. Exploratory MD Simulations for Initial Stability Assessment and Conformational Sampling

To assess the initial structural stability and characterize the dynamic behavior of the three functional conformations of the SARS-CoV-2 JN.1 Spike protein—closed, semi-closed, and open—100 ns molecular dynamics (MD) simulations were performed in triplicate for each conformational state. These simulations were designed to validate the stability of the homology-based models, identify dynamically active regions, and explore the accessible conformational space prior to extended simulations.

Trajectory analyses included evaluation of Cα root mean square deviation (RMSD), root mean square fluctuation (RMSF), radius of gyration (Rg), and solvent-accessible surface area (SASA). The closed conformation exhibited relatively stable RMSD profiles and lower fluctuation levels, consistent with a more compact architecture, whereas semi-closed and open conformations showed increased variability, particularly within the receptor-binding domain (RBD), reflecting enhanced domain mobility associated with partial or full RBD exposure.

Although these simulations revealed clear dynamic differences between conformational states, the 100 ns trajectories were not used directly for molecular docking analyses. Instead, they served as an exploratory stage to identify dominant dynamic features and guide the selection of representative conformations for extended simulations.

Based on RMSD behavior and conformational clustering using the GROMOS method [26], representative structures were selected from the most populated clusters during the final segment of each trajectory. Additional analyses of the 100 ns simulations, including RMSD profiles and filtered trajectories excluding highly flexible regions, are provided in Supplementary Figures S1 and S2.

These selected conformations were used as starting points for a second stage of 200 ns molecular dynamics simulations, enabling further conformational sampling and refinement of the structural ensembles. This stepwise strategy provides a consistent framework for capturing the dynamic behavior of the system while reducing bias associated with single-structure representations.

### 2.3. Structural Stability and Conformational Characterization of Spike JN.1

Extended 200 ns molecular dynamics simulations were performed to characterize the structural behavior of the SARS-CoV-2 JN.1 Spike protein across its closed, semi-closed, and open conformations. Multiple complementary descriptors, including root mean square deviation (RMSD), root mean square fluctuation (RMSF), radius of gyration (Rg), and solvent-accessible surface area (SASA), were analyzed to evaluate global stability, local flexibility, and conformational variability.

**Figure 2:**
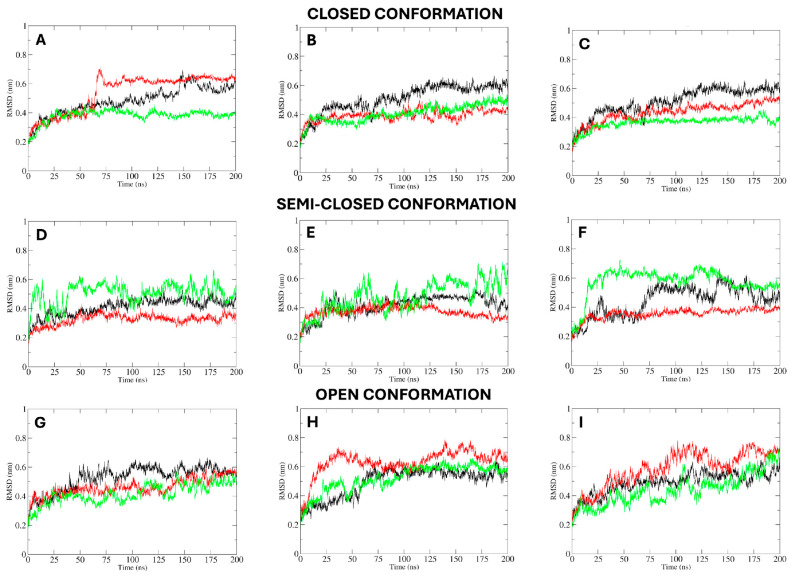
Root Mean Square Deviation (RMSD) profiles calculated for the Spike JN.1 protein along 200 ns molecular dynamics simulations. RMSD was computed over the Cα atoms of each protomer independently. Panels (**A**–**C**) correspond to the closed conformation, panels (**D**–**F**) to the semi-closed conformation, and panels (**G**–**I**) to the open conformation. For each conformational state, three independent replicas are shown. Chain A is represented in black, chain B in red, and chain C in green. RMSD profiles illustrate the overall structural stability and convergence behavior of each protomer across the different conformational states during extended molecular dynamics simulations.

RMSD profiles calculated over the Cα atoms of each protomer revealed an initial structural accommodation phase followed by sustained fluctuations throughout the simulations (Figure 2). While a gradual increase in RMSD was observed, particularly in semi-closed and open conformations, this behavior did not correspond to global structural destabilization. Instead, increased deviations were primarily associated with chains exhibiting RBD in the “up” position, reflecting large-amplitude domain motions characteristic of Spike functional dynamics [11,27].

To further distinguish between global structural rearrangement and localized flexibility, additional RMSD analyses were performed excluding highly mobile regions, including the RBD, N-terminal domain (NTD), and cleavage site (Supplementary Figure S3). These filtered trajectories exhibited markedly reduced deviations and stable profiles across all conformations and replicas, demonstrating that the structural core of the Spike trimer remains stable over the simulation timescale. Thus, the apparent RMSD drift observed in full-atom analyses is largely driven by intrinsically flexible domains rather than loss of global structural integrity [28,29].

**Figure 3:**
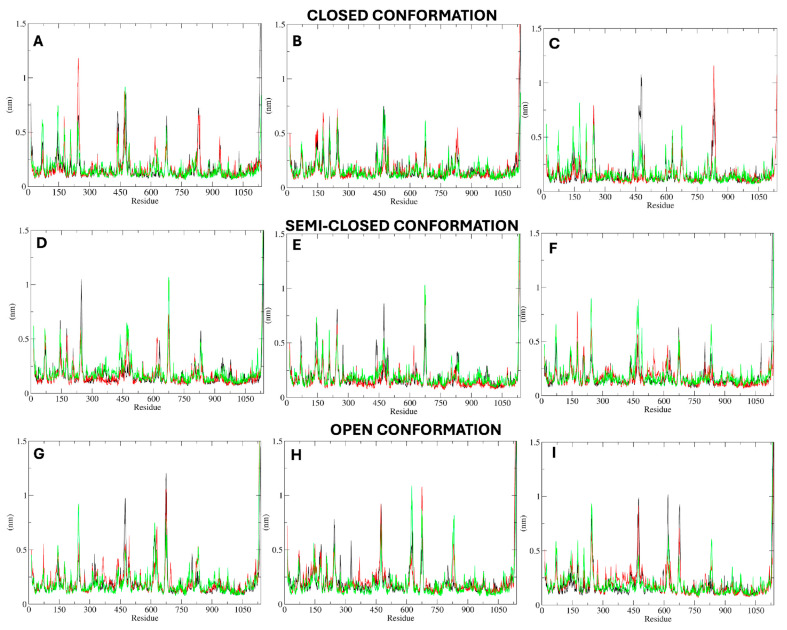
Per-residue root mean square fluctuation (RMSF) from molecular dynamics simulations of the JN.1 Spike protein in its three conformations: closed (top), semi-closed (middle), and open (bottom). Each plot displays the three protomer chains using the following colors: chain A (black), B (red), and C (green). (**A**–**C**) RMSF profiles of chains A, B, and C in the closed conformation, respectively; (**D**–**F**) RMSF profiles of chains A, B, and C in the semi-closed conformation, respectively; (**G**–**I**) RMSF profiles of chains A, B, and C in the open conformation, respectively. Peaks in flexibility are evident in mobile regions such as the RBD (residues from 319 to 541), with increased variation in the open conformation. These results support the use of representative structures for molecular docking studies.

Residue-level flexibility, assessed by RMSF, was calculated using the energy-minimized structure as a reference, confirming that most mobile regions correspond to surface-exposed loops, NTD, and RBD (Figure 3). Semi-closed and open conformations showed increased fluctuations in chains adopting the “up” configuration, consistent with enhanced conformational freedom and asymmetric trimer dynamics. These patterns were reproducible across replicas and highlight the dominant contribution of RBD mobility to overall Spike flexibility [30,31,32].

**Figure 4:**
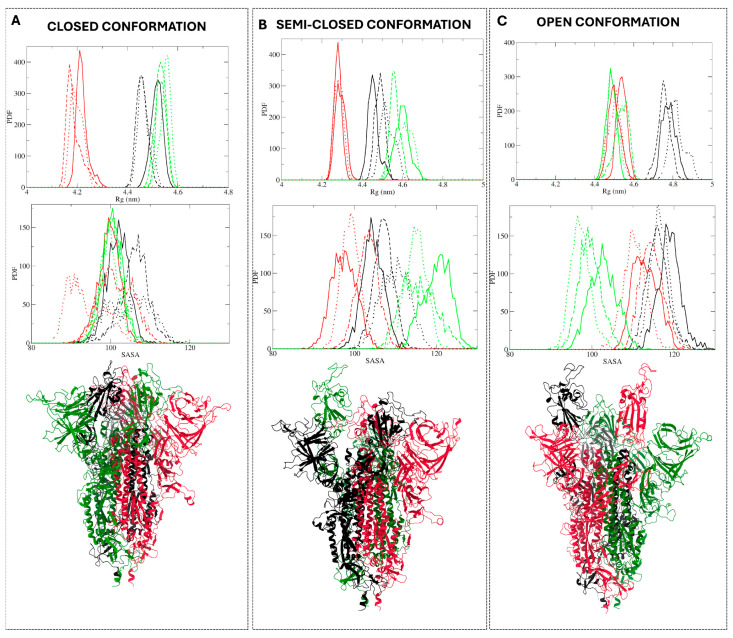
(**A**–**C**) Each panel corresponds to one functional state: closed (**A**), semi-closed (**B**), and open (**C**). Top graphs show the probability distribution function (PDF) of the radius of gyration (Rg) for each chain (A: black, B: red, C: green) across three MD replicas (solid, dashed, dotted lines). Middle graphs show the PDF of solvent-accessible surface area (SASA) for the same chains and replicas. Bottom panels depict the representative structures of the Spike trimer, colored by chain (A: black, B: red, C: green). The results demonstrate that open and semi-closed states exhibit greater structural flexibility and solvent exposure than the closed state, thereby supporting the functional conformational classification used in this study.

Global compactness analysis through radius of gyration (Rg) revealed clear differences among conformational states (Figure 4). Closed conformation exhibited narrower distributions centered at lower Rg values, consistent with a compact trimeric arrangement. In contrast, semi-closed and open conformations showed broader distributions and a shift toward higher Rg values, reflecting progressive structural expansion associated with RBD exposure and reduced interprotomer packing [33,34].

Consistently, SASA analysis demonstrated an increase in solvent exposure from closed to open conformations, with semi-closed states displaying intermediate behavior (Figure 4). These results support a model in which conformational transitions modulate the accessibility of functional surfaces without compromising the integrity of the trimeric core.

Overall, the combined RMSD, RMSF, Rg, and SASA analyses suggest that the JN.1 Spike protein maintains an overall preserved structural architecture alongside localized flexibility within structurally exposed regions, particularly in the RBD and NTD, consistent with the intrinsic conformational heterogeneity previously described for the Spike protein. These observations support the use of distinct conformational ensembles as a framework for subsequent ligand interaction analyses.

The selection of representative structures from the final segment of the trajectories was guided by the stabilization of the structural core and the convergence of global descriptors such as Rg and SASA. These conformations reflect the dominant dynamic regimes sampled during the simulations and provide a consistent structural basis for subsequent ligand interaction analyses [35,36].

### 2.4. Molecular Docking on the Spike Protein of the Wuhan Variant as a Reference System

To establish a structural reference for interpreting ligand recognition patterns, molecular docking analyses were performed on the Spike protein of the Wuhan strain. This system provides a well-characterized structural framework in which the interaction of flavonoids with the Spike protein has been previously explored [16].

In addition, the Wuhan Spike protein has been extensively characterized structurally and dynamically, making it a useful baseline for comparative evaluation of ligand interaction patterns across conformational states.

Docking calculations were conducted on the closed, semi-closed, and open conformations of the Wuhan Spike protein using structures derived from experimental data and prepared under consistent conditions. These analyses were intended to characterize baseline interaction patterns and the conformational dependence of ligand recognition.

In the closed conformation, both flavonoids were localized within relatively confined regions of the receptor-binding domain (RBD), consistent with the reduced accessibility of this structural state. H7R adopted a more localized interaction profile stabilized predominantly by polar and hydrogen-bond interactions, whereas F7G displayed a more spatially distributed interaction pattern across the cavity surface. Interestingly, several interacting positions identified in the Wuhan Spike protein correspond to residues later affected by mutations in emerging Omicron-derived variants, including positions within the 371–376 region and residue K417 of the RBD. Although the present study does not establish mutation-driven binding differences, these observations indicate that ligand-accessible regions identified in the Wuhan Spike overlap with structurally dynamic regions that remained relevant during subsequent variant evolution.

**Figure 5:**
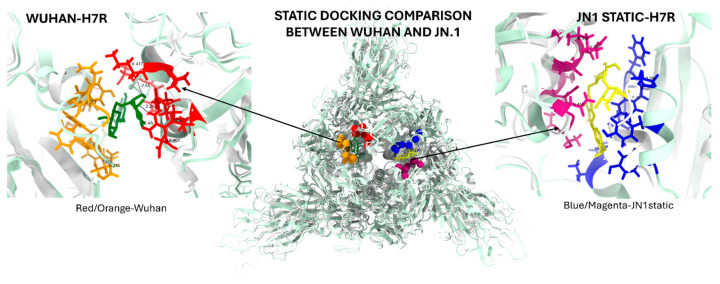
Comparative static docking analysis of H7R in the closed conformations of the SARS-CoV-2 Wuhan and JN.1 Spike proteins. Representative docking poses obtained from static docking analyses are shown superimposed within the S1 subunit of each Spike structure. The Wuhan Spike protein is represented as a white cartoon, whereas the JN.1 static model is shown in light green. Representative interaction residues associated with the Wuhan complex are shown in red and orange, while residues associated with the JN.1 static complex are shown in blue and magenta. The H7R ligand is represented in green for the Wuhan complex and in yellow for the JN.1 static complex. Central structural mapping illustrates the spatial distribution of representative ligand poses across both variants, while magnified views highlight differences in ligand orientation and local interaction environments. The comparison reveals partially overlapping but spatially distinct ligand-interaction regions, indicating that structural context influences the location and distribution of docking poses even under equivalent static docking conditions.

Transition to semi-closed and open conformations resulted in increased exposure of the RBD and the emergence of more extended binding regions. Under these conditions, both ligands explored broader interaction surfaces, with F7G exhibiting greater spatial adaptability and H7R maintaining a comparatively more localized interaction network. Comparative visualization of representative static docking poses between the Wuhan and JN.1 Spike proteins revealed partially overlapping interaction regions together with differences in ligand orientation and spatial distribution (Figure 5). These differences suggest that structural context influences the accessible ligand interaction landscape even under equivalent static docking conditions.

Overall, these results indicate that ligand recognition in the Wuhan Spike protein is strongly influenced by conformational state, with more open conformations enabling expanded interaction patterns. The observed interaction profiles provide a structural and methodological reference framework for interpreting ligand recognition landscapes in the JN.1 variant and for evaluating the impact of conformational sampling on docking outcomes.

### 2.5. Molecular Docking on Dynamically Derived Conformations of Spike JN.1

To evaluate ligand recognition in the JN.1 variant, molecular docking analyses were performed on representative conformations obtained from molecular dynamics simulations. In contrast to the Wuhan reference system, docking was conducted exclusively on structures derived from conformational sampling (after 200 ns), enabling evaluation of ligand interactions within dynamically generated structural ensembles.

**Figure 6:**
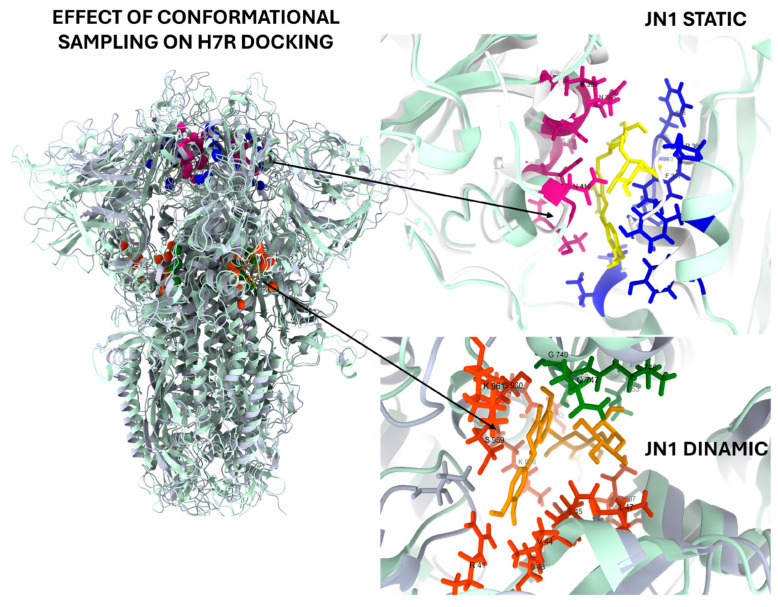
Effect of conformational sampling on H7R docking in the SARS-CoV-2 JN.1 Spike protein. Representative H7R docking poses obtained from static and dynamically sampled closed conformations of the JN.1 Spike protein are shown mapped onto the Spike trimer. The static JN.1 structure is represented as a light-green cartoon, whereas the dynamically sampled JN.1 conformation is shown in gray. In the upper panel, H7R docked to the static JN.1 structure is represented in yellow, with associated interaction residues shown in blue and magenta. In the lower panel, H7R docked to the dynamically sampled JN.1 conformation is represented in gold, with interaction residues shown in orange and green. Magnified views illustrate differences in ligand orientation and accessible interaction regions between static docking and docking performed on structures derived from molecular dynamics simulations. The comparison demonstrates that conformational sampling modifies the location and accessibility of ligand-interaction regions and reveals additional recurrent docking orientations not observed in the corresponding static structure.

Representative structures were selected from the most populated clusters obtained during the final segment of the 200 ns simulations (final 75 ns of each replica) for each conformational state. These structures capture the dominant conformational regimes sampled during the simulations and provide a consistent framework for analyzing ligand interaction patterns. To evaluate the influence of structural context on ligand recognition, comparative visual analyses were performed between static docking poses obtained from the Wuhan and JN.1 Spike proteins, as well as between static and dynamically sampled JN.1 conformations (Figure 5 and Figure 6). These comparisons revealed that conformational sampling expanded the accessible interaction landscape and generated additional recurrent docking orientations not observed in static structures alone. These observations directly address the central objective of the study by demonstrating that conformational sampling modifies the location, accessibility, and recurrence of ligand-interaction regions. Comparative structural visualizations focused on H7R as a representative ligand due to its more spatially localized docking distributions across conformational states, which facilitated direct comparison between static and dynamically sampled structures.

To further evaluate the structural recurrence of docking outcomes, RMSD-based clustering (2 Å cutoff) was performed for docking poses across all JN.1 conformational states (Supplementary Figures S6–S11). Structural mapping of dominant clusters revealed that the most populated docking solutions corresponded to recurrent spatial interaction modes rather than isolated lowest-energy poses (Figure S4). In several cases, secondary clusters occupied either overlapping orientations within the same ligand-accessible region or alternative recurrent regions within the S1 subunit, supporting the structural consistency of the observed ligand recognition patterns.

**Figure 7:**
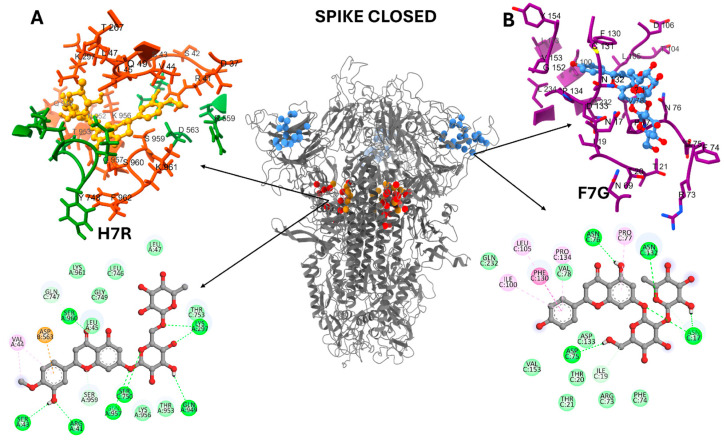
Docking results of hesperitin-7-O-rutinoside (H7R) and flavanone-7-O-glucoside (F7G) on the representative Spike JN.1 structure obtained from clustering of the last segment of the 200 ns molecular dynamics simulations. The central panel shows the global binding regions on the Spike trimer, where interaction hotspots are highlighted as spheres (red and yellow for H7R; blue for F7G). The left panel (**A**) presents the three-dimensional binding pose of H7R within the receptor-binding domain (RBD), together with a two-dimensional interaction map indicating the residues involved in ligand recognition. The right panel (**B**) shows the corresponding three-dimensional binding pose and interaction map for F7G. Residues are colored according to interaction type, illustrating hydrogen bonding (green), hydrophobic contacts (magenta) and van der Waals contacts (light green) stabilizing each ligand within the dynamically equilibrated binding pocket.

In the closed conformation, H7R exhibited a localized binding mode within a confined region, stabilized by a network of polar interactions involving residues such as SER43, ARG41, and GLN949. In contrast, F7G displayed a broader interaction profile, engaging multiple residues across the cavity surface through distributed polar and hydrophobic contacts. This difference suggests distinct recognition strategies, with H7R favoring directional interactions and F7G adapting to geometric constraints through interaction dispersion (Figure 7).

**Figure 8:**
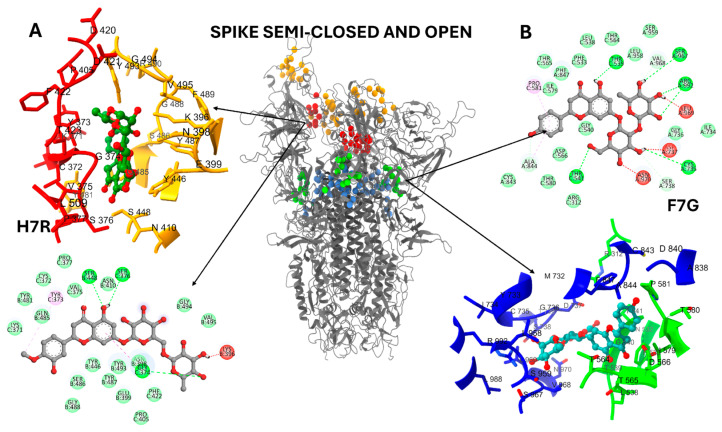
Docking interaction maps of flavonoids on the semi-closed and open conformations of the SARS-CoV-2 Spike JN.1 protein, obtained from clustering of extended 200 ns molecular dynamics simulations. The central panel shows the spatial distribution of ligand-binding regions mapped onto the Spike trimer. (**A**) Representative three-dimensional binding pose and two-dimensional interaction map of H7R. (**B**) Representative three-dimensional binding pose and two-dimensional interaction map of F7G. Interaction maps indicate hydrogen bonds (green), van der Waals contacts (light green), and hydrophobic interactions (magenta) involved in ligand stabilization.

In semi-closed and open conformations, increased exposure of the RBD generated larger and more heterogeneous binding regions. Under these conditions, both ligands occupied overlapping cavities but maintained distinct interaction patterns. H7R remained associated with more localized anchoring sites, forming specific hydrogen bonds and aromatic interactions, whereas F7G adopted extended binding modes involving a wider range of residues, including polar, hydrophobic, and electrostatic contacts (Figure 8).

These observations indicate that ligand recognition in JN.1 is modulated by conformational variability and that dynamically derived structures reveal interaction patterns not accessible in more constrained conformational states. Importantly, supplementary structural clustering analyses demonstrate that these interaction patterns were supported by recurrent docking populations across independent docking runs, reinforcing that the reported binding modes reflect structurally consistent interaction tendencies rather than isolated docking solutions.

### 2.6. Analysis of Docking Score Distributions and Ligand Interaction Trends

To further assess the consistency of docking results, the distribution of docking scores obtained from 100 independent runs was analyzed for each conformational state (Supplementary Figure S4). In all cases, docking scores were concentrated within a narrow energy range, indicating reproducible sampling of ligand–protein interaction modes across independent simulations.

Consistent with these observations, clustering analysis revealed that a limited number of interaction modes accounted for most docking poses (Table 1), supporting the presence of recurrent and structurally consistent binding patterns.

Docking scores obtained using AutoDock Vina were analyzed comparatively to evaluate ligand–protein interaction patterns across conformational states. Because docking scores were concentrated within a relatively narrow energy range, structural recurrence and pose distribution were considered more informative descriptors of dominant interaction behavior than absolute score minimization. Consistently, clustering analysis demonstrated that the most populated interaction modes did not necessarily coincide with the lowest-energy bins, highlighting the importance of recurrent docking patterns over isolated low-energy poses.

Across all systems, both ligands exhibited conformationally dependent interaction patterns. F7G consistently displayed high pose recurrence across conformations, reflecting its ability to establish distributed interaction networks across structurally diverse environments. In contrast, H7R showed greater variability in pose distribution, consistent with its preference for more spatially defined interaction regions.

Docking analyses performed on dynamically derived JN.1 conformations revealed changes in pose distribution and interaction patterns compared to homology-based structures, indicating that conformational sampling influences the location, accessibility, and recurrence of ligand-interaction regions. These differences were further supported by comparative structural visualizations between static and dynamically sampled conformations (Figure 6), where dynamically derived structures revealed additional recurrent docking orientations and broader spatial distributions. Comparative overlays showed that docking on dynamically sampled conformations enabled ligand occupation of structurally accessible regions not observed in the corresponding static JN.1 model.

These differences were particularly evident in semi-closed and open conformations, where increased structural variability enabled a broader range of ligand interactions. These observations do not rely on absolute differences in docking scores but instead reflect consistent shifts in interaction patterns and pose distributions across conformational states. The combined analysis of docking scores, pose frequencies, and interaction patterns supports the use of dynamically derived conformations to complement static docking approaches when describing ligand–protein interactions in flexible systems such as the SARS-CoV-2 Spike protein.

## 3. Discussion

The continuous emergence of SARS-CoV-2 variants characterized by increased transmissibility and immune escape highlights the need to understand how conformational heterogeneity and structural dynamics of the Spike protein influence ligand recognition. In this study, we combined homology modeling, hierarchical molecular dynamics (MD) simulations, and molecular docking to characterize the conformational behavior of the Spike protein from the JN.1 subvariant and to evaluate ligand–protein interaction patterns using two flavonoids, H7R and F7G, as molecular probes.

Due to the absence of experimentally resolved structures for JN.1 at the time of this work, homology modeling based on closely related BA.2 templates was employed as an initial step [37]. While these models preserved the canonical trimeric architecture of the Spike protein, subsequent MD simulations indicate that static structures alone do not fully capture the conformational variability of this system. Analyses based on RMSD, RMSF, radius of gyration (Rg), and solvent-accessible surface area (SASA) consistently showed that the closed, semi-closed, and open conformations correspond to distinct dynamic regimes. Importantly, the observed deviations in RMSD are largely influenced by localized flexibility within the receptor-binding domain (RBD) and N-terminal domain (NTD), rather than global structural instability, as supported by analyses excluding highly flexible regions (Supplementary Figures S1 and S2). To further assess residue-level flexibility, RMSF values were mapped onto the Spike structure for each conformational state (Supplementary Figure S5), allowing visualization of the spatial distribution of fluctuations.

However, rather than indicating fundamentally different stability regimes, these observations reflect expected structural rearrangements associated with functional transitions between conformational states.

The hierarchical MD strategy employed in this study—comprising an initial exploratory stage (100 ns), conformational clustering, and extended simulations (200 ns)—enabled the selection of representative conformations for downstream analysis. The 100 ns simulations were not used directly for docking but served to identify dominant dynamic features and guide conformational sampling [12,29,30,35]. The extended simulations provided more stable and consistent structural descriptors across replicas; however, convergence in large, flexible systems such as the Spike trimer remains inherently limited and should be interpreted cautiously. Therefore, the selected conformations should be considered dynamically representative states rather than fully converged ensembles.

Docking analyses revealed that ligand interaction patterns depend on the conformational state and structural context of the receptor. These observations directly address the central objective of this study, demonstrating that conformational sampling modifies the location, accessibility, and recurrence of ligand-interaction regions compared with static structural models. Structures derived from molecular dynamics simulations exhibited broader and more accessible interaction regions than static conformations, reflecting differences in conformational sampling, solvent exposure, and the dynamic accessibility of ligand-recognition landscapes [13,32,38,39].

The use of AutoDock Vina as a scoring and sampling framework is supported by its demonstrated performance in reproducing ligand binding modes and identifying consistent interaction patterns across diverse benchmark datasets, including PDBbind, where typical deviations in predicted binding energies are within the expected range for empirical scoring functions [40]. While absolute binding free energies cannot be directly inferred, Vina provides a consistent and reproducible framework for comparative analysis of ligand–protein interaction patterns under equivalent conditions [40].

Within this framework, F7G and H7R exhibited distinct interaction behaviors across conformational states. F7G showed recurrent binding poses across different structural contexts, suggesting a higher tolerance to variations in pocket geometry. In contrast, H7R displayed a stronger dependence on more exposed conformations, where interaction networks could be established within more accessible regions [15,41].

These differences do not necessarily imply stronger binding in thermodynamic terms but rather reflect distinct modes of interaction under varying structural conditions [39,41].

Previous computational studies have reported the interaction of flavonoids such as H7R and F7G with the SARS-CoV-2 Spike protein using docking-based approaches, identifying binding regions within the S1 subunit and the receptor-binding domain [39,42,43]. Collectively, these studies support the ability of such compounds to interact with structurally accessible regions of the Spike protein and reinforce their use as molecular probes to explore ligand-recognition patterns. In this context, interaction modes identified in the present study are consistent with previously reported binding tendencies, supporting the structural plausibility of the observed docking poses.

The use of the Wuhan Spike protein as a reference system provided a structurally characterized baseline for comparison; however, it should not be considered a validated ligand-binding system. Importantly, the comparison between the Wuhan and JN.1 variants involves structures obtained under different levels of structural refinement, as the JN.1 models were subjected to extensive molecular dynamics simulations, whereas the Wuhan structures correspond to crystallographic models. Therefore, differences observed between both systems may arise from a combination of sequence variation and structural relaxation effects.

Extensive molecular dynamics studies of the Wuhan Spike protein have demonstrated its intrinsic conformational heterogeneity, including transitions between closed, semi-open, and open states driven by receptor-binding domain (RBD) dynamics [10,12,29,30]. These findings indicate that conformational flexibility is an inherent property of the Spike protein rather than a variant-specific feature. Consequently, the present work does not aim to establish direct mutation-driven differences between variants, but rather to evaluate how conformational sampling and structural context influence ligand recognition patterns in a highly flexible system.

Importantly, the flavonoids analyzed in this study are not proposed as direct antiviral inhibitors but are used as molecular probes to explore how conformational variability influences ligand recognition. While previous studies have suggested their potential interaction with the Spike protein [16,17,18,19], there is currently no direct experimental evidence confirming their binding modes in isolation to our knowledge. Consequently, the interaction patterns reported here should be interpreted as model-dependent observations rather than experimentally validated binding mechanisms.

From a methodological perspective, comparative analyses between static and dynamically sampled conformations indicate that conformational sampling modifies the location, accessibility, and recurrence of ligand-interaction regions. Comparative structural overlays demonstrated that molecular dynamics-derived structures expose alternative ligand-accessible regions and recurrent docking orientations that remain inaccessible in structurally constrained static models. While static docking provided a useful approximation of ligand-accessible regions within structurally constrained conformations, ensemble-based docking on dynamics-informed structures captured greater spatial diversity and broader interaction distributions. These observations support the incorporation of conformational sampling strategies when evaluating highly flexible proteins such as the SARS-CoV-2 Spike trimer.

Several limitations should be considered. First, the absence of experimentally resolved ligand–Spike complexes limits the validation of docking poses. Second, the use of large docking grids and the lack of predefined binding sites introduce uncertainty in the identification of preferred interaction regions. Third, although extended MD simulations improve conformational sampling, full convergence of such a large and flexible system cannot be guaranteed. Finally, docking scores were interpreted in a comparative context, and small differences in energy values should be considered within the intrinsic uncertainty of empirical scoring functions. Although docking scores were distributed within a relatively narrow energy range (Supplementary Figure S4), clustering analysis indicates that the most populated interaction modes do not necessarily correspond to the lowest-energy poses. This highlights the importance of structural recurrence and conformational sampling over absolute scoring when interpreting docking results, particularly in highly flexible systems such as the SARS-CoV-2 Spike protein.

Overall, this study provides a computational framework to explore how conformational heterogeneity influences ligand recognition in the SARS-CoV-2 Spike protein. Rather than establishing definitive binding mechanisms or variant-specific affinity differences, the present work highlights the methodological importance of incorporating conformational sampling and structural ensembles when evaluating ligand-recognition behavior in highly flexible viral proteins such as the SARS-CoV-2 Spike trimer.

## 4. Materials and Methods

### 4.1. Structural Modeling of the Spike Protein JN.1 Variant

The three-dimensional structures of the Spike protein of the SARS-CoV-2 JN.1 variant were generated by homology modeling using SWISS-MODEL [23]. The amino acid sequence (NCBI: WRK13149.1) reported in early 2024 was aligned against crystallographic templates of the Omicron BA.2 variant, including PDB entries 8D55, 8D56 [24] and 8DM7 [25] with a sequence identity of 97.08%. The final models comprised residues 14 to 1126.

Three conformational states—closed (all RBDs down), semi-closed (one RBD up), and open (two RBDs up)—were generated by selecting template structures representing each state during the modeling process. Each conformation was built independently to preserve the structural arrangement of the receptor-binding domains (RBD) in their corresponding orientations. Model quality was assessed using GMQE and QMEAN scoring functions provided by SWISS-MODEL, ensuring structural reliability for subsequent simulations.

### 4.2. Reference Structures for Wuhan Variant

Crystallographic models corresponding to the closed (PDB: 6VXX), semi-closed (PDB: 6VSB), and open (PDB: 6X2B) [9,27,44] states of the Wuhan-Hu-1 Spike protein were retrieved from the CHARMM-GUI COVID-19 Proteins Library [45]. These models are complete and energy-minimized using 100 steps of the Steepest Descent method in CHARMM [46].

### 4.3. Molecular Dynamics Simulations

Molecular dynamics (MD) simulations were performed to evaluate the structural stability, conformational flexibility, and dynamic behavior of the SARS-CoV-2 JN.1 Spike protein model in its three functional states: closed, semi-closed, and open.

All simulations were carried out using GROMACS version 2019.2, applying the CHARMM36m [47] force field, which is well suited for proteins with flexible regions and transmembrane domains. System setup was performed using CHARMM-GUI [45], including protonation state assignment. All systems were solvated in a cubic water box with a 10 Å buffer using TIP3P water molecules and neutralized with K^+^ and Cl^−^ ions at a final salt concentration of 0.15 M. Simulations were run under physiological conditions (310 K, 1 atm, pH 7). All systems were first equilibrated using the NVT ensemble and then simulated under the NPT ensemble.

For each conformational state (closed, semi-closed, and open), three independent 100 ns production simulations were performed under periodic boundary conditions. A 4 fs integration time step was employed by applying Hydrogen Mass Repartitioning (HMR) [48], a strategy previously validated for protein simulations that maintains stable integration without introducing significant artifacts in structural descriptors such as RMSD and RMSF, and all covalent bonds involving hydrogen atoms were constrained using the LINCS algorithm. Long-range electrostatic interactions were treated using the Particle Mesh Ewald (PME) method.

Representative structures were selected based on RMSD behavior and conformational clustering using the GROMOS algorithm with a cutoff of 0.25 nm applied to Cα atoms, allowing identification of the most populated conformational states during the final 50 ns of each trajectory. The representative structure from the dominant cluster was selected for each replica. These equilibrated structures were used as starting points for a second stage of extended MD simulations of 200 ns, again performed in triplicate for each conformational state.

From these extended simulations (200 ns), the last 75 ns of each replica were extracted and combined into a single trajectory, from which a final representative structure was selected using the GROMOS algorithm with a cutoff of 0.25 nm applied to Cα atoms (Supplementary Table S1). This hierarchical MD strategy ensured that the conformations used for subsequent analyses and molecular docking corresponded to well-equilibrated and dynamically representative states of the JN.1 Spike protein, thereby improving the reliability of structure-based interaction analyses.

Trajectory analyses were performed to characterize structural stability, residue-level flexibility, global compactness, and solvent accessibility of the Spike protein across conformations. RMSD, RMSF, radius of gyration (Rg), and solvent-accessible surface area (SASA) were calculated using GROMACS built-in analysis tools.

RMSD calculations were performed on Cα atoms for each chain of the Spike trimer to assess global structural stability and convergence. RMSD profiles were first evaluated over the initial 100 ns simulations to confirm equilibration trends and subsequently over the extended 200 ns simulations to verify long-term stability, particularly for the semi-closed and open conformations.

RMSF values were computed on a per-residue basis using Cα atoms to quantify local flexibility. RMSF analyses focused on the equilibrated regions of the trajectories, specifically the last 75 ns of each replica, enabling identification of dynamically relevant regions such as the receptor-binding domain (RBD) and flexible loop segments.

The radius of gyration (Rg) was calculated independently for each chain to evaluate global compactness and conformational expansion associated with RBD rearrangements. Rg distributions were compared across replicas and conformations to assess differences between closed, semi-closed, and open states.

SASA calculations were performed to quantify solvent exposure and conformational accessibility. SASA profiles provided an independent structural metric supporting the classification of conformational states, complementing definitions based on RBD orientation.

All analyses were conducted on equilibrated portions of the trajectories, ensuring that reported structural descriptors reflect dynamically stable and representative conformations used for subsequent molecular docking studies. All trajectory analyses were performed using GROMACS version 2024.5 built-in tools and custom Linux scripts. Visualization and figure generation were conducted using Visual Molecular Dynamics (VMD) version 1.9.3 [49], UCSF Chimera version 1.19 [50], ChimeraX version 1.10.1 [51], and Discovery Studio Visualizer version 2024 [52].

Convergence of the simulations was assessed based on the stabilization of RMSD profiles and the consistency of structural descriptors (RMSF, Rg, and SASA) across independent replicas. While complete convergence cannot be strictly ensured for large systems such as the Spike trimer, these criteria support the use of the selected conformations as representative states for subsequent analyses.

### 4.4. Ligand Preparation

Two natural flavonoids, identified as hesperitin 7-O-rutinoside (H7R) and flavanone-7-O-glucoside (F7G) (PubChem IDs: 10621 and 442428, respectively), were selected for docking experiments. Ligand structures were optimized and prepared in PDBQT format as implemented on UCSF Chimera [50].

### 4.5. Molecular Docking Protocol

Molecular docking was performed using AutoDock Vina v1.2.5 [40]. For the Wuhan variant, a blind docking approach was applied using a grid box of 175 × 185 × 105 Å centered at x = 11.60, y = −4.64, z = 229.307. For the JN.1 variant, a grid box of 119 × 152 × 145 Å centered at x = −63.77, y = 1.31, z = 7.97 was used. The docking search space was defined using a blind docking strategy designed to encompass the S1 subunit, including both the N-terminal domain (NTD) and receptor-binding domain (RBD), thereby allowing broad exploration of ligand-accessible regions without imposing predefined binding sites (Supplementary Figure S12). For each receptor-ligand pair (three conformations × two ligands), 100 independent docking runs were performed.

The use of 100 independent docking runs per system was intended to enhance sampling of ligand poses and identify recurrent interaction patterns, rather than to provide statistical measures of binding affinity. Structures with RMSD < 2 Å were clustered to identify the most populated binding poses and to estimate average docking scores. Clusters corresponding to equivalent binding regions across different Spike chains were merged when visual inspection confirmed spatial overlap within the same binding site. This approach accounts for the trimeric symmetry of the Spike protein and avoids artificial fragmentation of equivalent interaction modes. Although grid dimensions differed between systems due to variations in structural orientation, the search space was consistently defined to encompass the S1 subunit, including the NTD and RBD, thereby enabling comparable and unbiased exploration of ligand-accessible regions across conformations and variants (Supplementary Figure S12). All water molecules and ions were removed prior to docking calculations, and only the protein structure was considered for ligand binding analysis. Docking scores obtained from AutoDock Vina were interpreted qualitatively, as the scoring function provides approximate estimates of interaction tendencies rather than absolute binding free energies.

### 4.6. Interaction Analysis

Residues within 5 Å of the docked ligands were identified using UCSF Chimera [50]. Interaction maps were generated with Discovery Studio Visualizer [52] to visualize conserved and unique binding pockets across conformations and variants (Figure 5, Figure 6 and Figure 7). Sequence alignments were performed using Clustal Omega version 1.2.4 [9] (to evaluate the impact of JN.1-specific mutations and deletions on ligand binding).

## 5. Conclusions

In this study, we applied a hierarchical computational framework integrating homology modeling, molecular dynamics simulations, and ensemble docking to investigate how conformational sampling and structural context influence ligand recognition in the SARS-CoV-2 JN.1 Spike protein. The results demonstrate that different conformational states of the Spike trimer generate distinct ligand-accessible interaction landscapes, particularly as receptor-binding domain (RBD) exposure increases from closed to more open conformations. Comparative analyses between static and dynamically sampled structures showed that conformational sampling expands the spatial distribution of accessible interaction regions and reveals additional recurrent docking poses not observed in static structures alone. Static docking analyses performed on the Wuhan and JN.1 Spike proteins identified partially overlapping but spatially distinct ligand-interaction regions, indicating that structural context influences ligand orientation and pose distribution even under equivalent docking conditions. However, these comparisons should be interpreted cautiously, since the present study does not establish mutation-driven differences in binding affinity or validated functional binding mechanisms.

The distinct behaviors observed for H7R and F7G further highlight the combined influence of ligand geometry and receptor conformational heterogeneity on docking outcomes. H7R exhibited more spatially localized interaction patterns that facilitated comparative structural analyses, whereas F7G displayed broader pose dispersion and more extended interaction landscapes across dynamically sampled conformations.

From a methodological perspective, static docking provided a useful initial approximation of ligand-accessible regions within structurally constrained conformations, whereas docking performed on dynamics-informed structural ensembles captured a broader diversity of recurrent interaction modes. These observations support the incorporation of conformational sampling and ensemble-based docking strategies when investigating highly flexible viral proteins such as the SARS-CoV-2 Spike trimer. Rather than defining definitive antiviral mechanisms or absolute binding affinities, this work provides an exploratory computational framework for evaluating ligand-recognition behavior in structurally dynamic viral systems.

## Figures and Tables

**Table 1:** Summary of docking score trends and pose distributions for flavonoids F7G and H7R across different conformational states of the SARS-CoV-2 Spike protein. Docking scores (kcal/mol) and pose frequencies (%) were obtained from 100 independent docking runs for each conformational state (closed, semi-closed, and open) and structural model, including the Wuhan Spike protein, JN.1 homology models, and representative conformations derived from molecular dynamics simulations (100 ns and 200 ns). Docking scores are reported as mean ± standard deviation of sampled poses and are interpreted qualitatively, as AutoDock Vina scoring functions provide approximate estimates of relative interaction tendencies rather than absolute binding free energies. Differences in docking scores within a narrow range should therefore not be considered quantitatively significant. Pose frequency (%) represents the proportion of docking poses belonging to the most populated binding mode and is used as an indicator of recurrent interaction patterns across conformational states. Structural mapping of dominant and secondary docking clusters for JN.1 conformations is provided in Supplementary Figures S6–S11, illustrating whether recurrent docking populations correspond to shared or distinct ligand-accessible regions. The 100 ns-derived structures correspond to an exploratory stage, while the 200 ns-derived conformations represent the primary basis for comparative analysis.

	Wuhan Variant				JN.1 Variant Homology				JN.1 Variant Clustering 100 ns				JN.1 Variant Clustering 200 ns			
	F7G (%)	Energy	H7R (%)	Energy	F7G (%)	Energy	H7R (%)	Energy	F7G (%)	Energy	H7R (%)	Energy	F7G (%)	Energy	H7R (%)	Energy
Closed	51	−9.79 ± 0.33	35	−10.24 ± 0.25	52	−9.95 ± 0.26	33	−10.07 ± 0.07	78	−9.38 ± 0.05	75	−9.66 ± 0.56	87	−9.63 ± 0.02	49	−9.79 ± 0.24
Semi-Closed	25	−9.70 ± 0.62	34	−9.57 ± 0.27	40	−9.90 ± 0.20	53	−9.89 ± 0.10	72	−9.57 ± 0.19	49	−10.73 ± 0.10	32	−9.97 ± 0.36	32	−9.94 ± 0.08
Open	20	−10.19 ± 0.15	16	−10.49 ± 0.42	31	−9.13 ± 0.55	96	−9.81 ± 0.32	76	−9.43 ± 0.06	88	−10.2 ± 0.25	50	−9.74 ± 0.17	47	−10.04 ± 0.13

## Data Availability

The original contributions presented in this study are included in the article. Further inquiries can be directed to the corresponding author.

## Supplementary Materials

The following supporting information can be downloaded at https://www.mdpi.com/article/10.3390/ijms27156624/s1.

## Abbreviations

The following abbreviations are used in this manuscript:

SARS-CoV-2	Severe Acute Respiratory Syndrome Coronavirus 2
S	Spike
RBD	Receptor-Binding Domain
NTD	N-terminal domain
H7R	Hesperitin 7-O-Rutinoside
F7G	Flavanone-7-O-Glucoside
ACE2	Angiotensin Converting Enzyme 2
MD	Molecular Dynamics
GMQE	Global Model Quality Estimation
QSQE	Quaternary Structure Quality Estimate
RMSD	Root Mean Square Deviation
RMSF	Root Mean Square Fluctuation
Rg	Radius of Gyration
