# Supplementary material for: Flavonoids as Structural Probes Reveal Conformationally Dependent Ligand Recognition in the SARS-CoV-2 JN.1 Spike Protein
Source: Int J Mol Sci. 2026 Jul 24;27(15):6624. doi: 10.3390/ijms27156624 (PMC13465250; doi:10.3390/ijms27156624)
Supplement: Supplementary file 1 [file ijms-27-06624-s001.zip › ijms-4233207-supplementary.pdf]

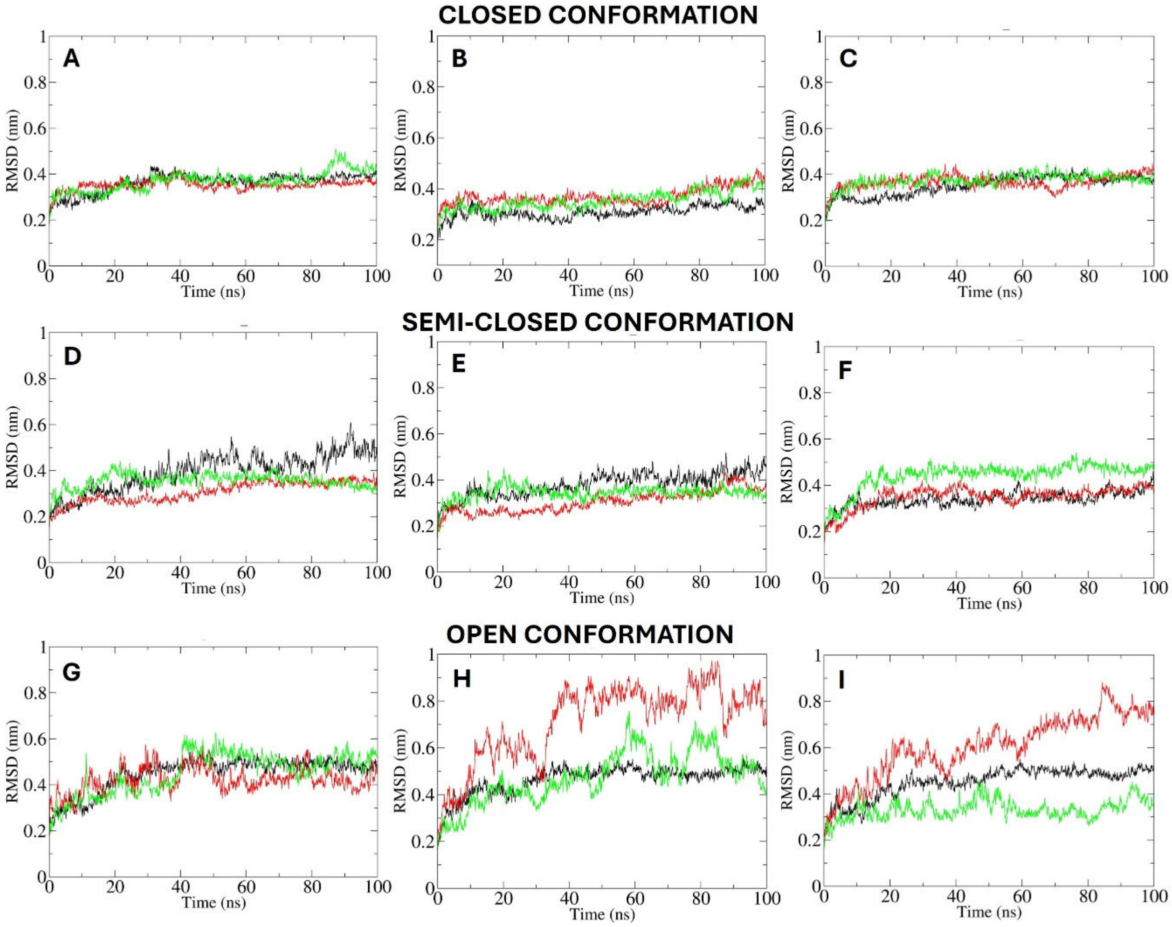

Figure S1.

Root mean square deviation (RMSD) profiles of the SARS-CoV-2 JN.1 Spike protein during the initial 100 ns molecular dynamics simulations. Each panel corresponds to an independent replica across the three conformational states: closed (A–C), semi-closed (D–F), and open (G–I). RMSD was calculated using Cα atoms for each protomer chain (A: black, B: red, C: green). While an initial stabilization phase is observed, higher deviations are present in semi-closed and open conformations, primarily associated with flexible domains such as the receptor-binding domain (RBD) and N-terminal domain (NTD).

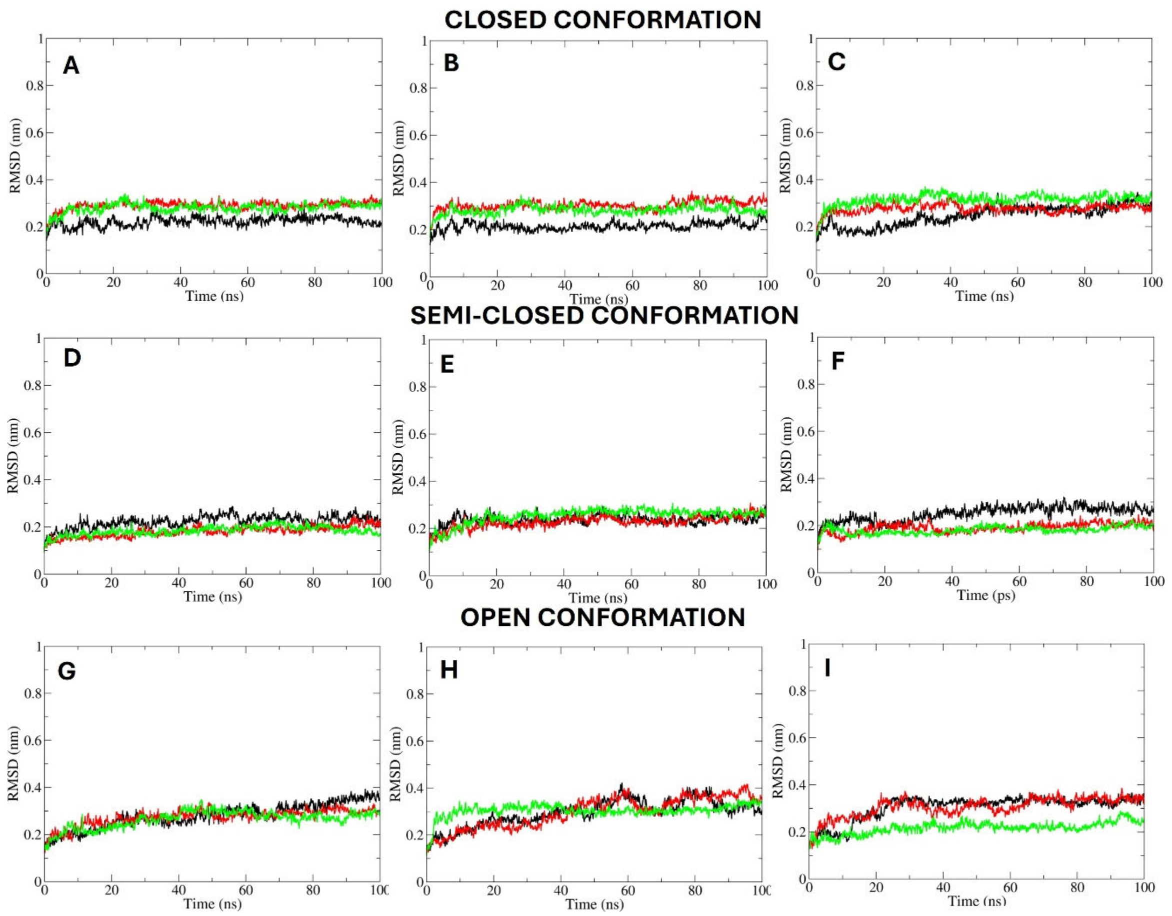

Figure S2.

RMSD profiles of the SARS-CoV-2 JN.1 Spike protein during 100 ns molecular dynamics simulations calculated after excluding highly flexible regions, including the receptor-binding domain (RBD), N-terminal domain (NTD), and loop regions. Each panel corresponds to independent replicas for closed (A–C), semi-closed (D–F), and open (G–I) conformations. RMSD values were calculated using Cα atoms for each protomer chain. Compared to full-structure RMSD (Figure S1), the filtered trajectories exhibit reduced deviations and improved stability, indicating that the observed structural fluctuations are primarily driven by localized flexibility rather than global structural instability.

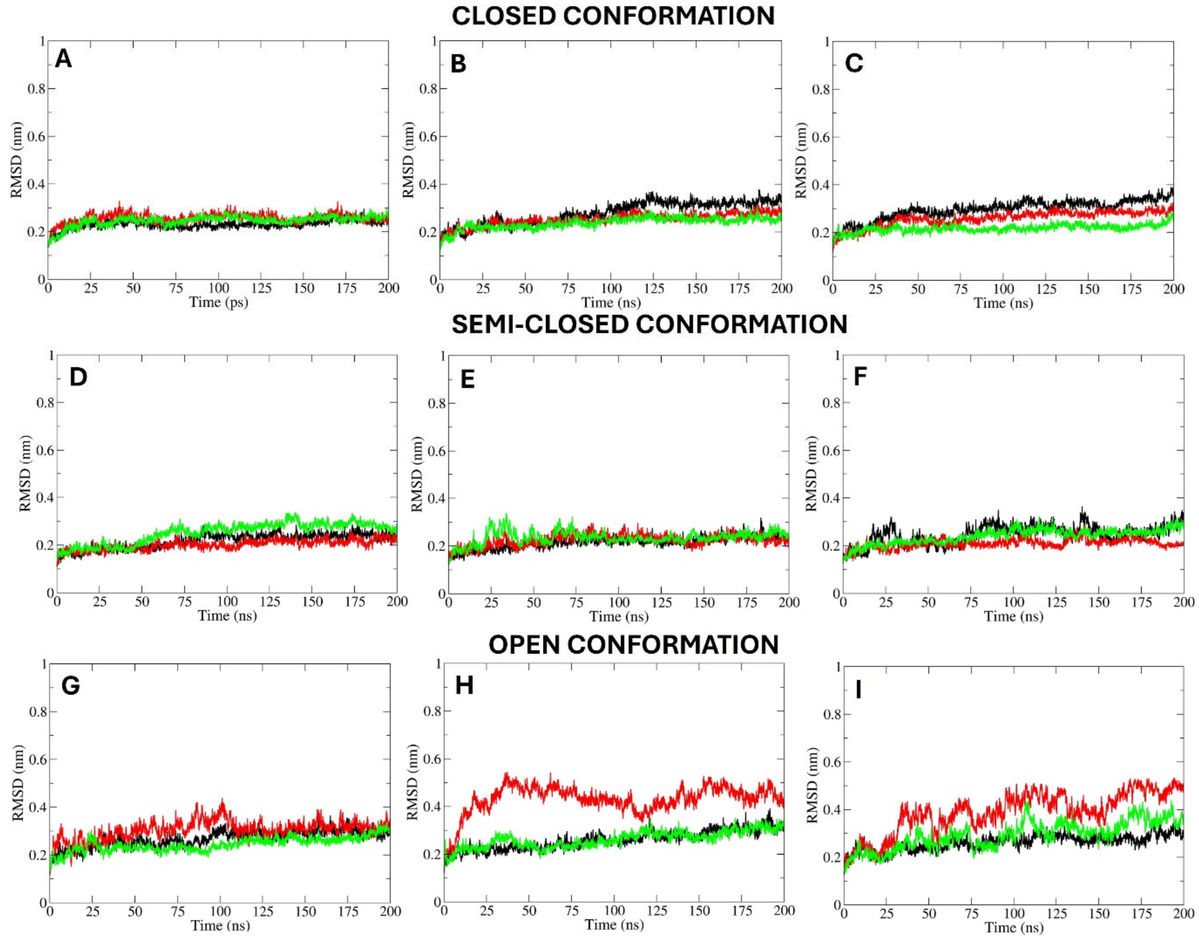

Figure S3.

RMSD profiles of the SARS-CoV-2 JN.1 Spike protein during extended 200 ns molecular dynamics simulations calculated after excluding highly flexible regions, including the receptor-binding domain (RBD), N-terminal domain (NTD), and loop regions. Each panel corresponds to independent replicas for closed (A–C), semi-closed (D–F), and open (G–I) conformations. RMSD values were calculated using Cα atoms for each protomer chain. Compared to the full-structure RMSD profiles shown in Figure 2, the filtered trajectories exhibit reduced deviations and improved stability across all conformations, indicating that the observed fluctuations are primarily associated with localized flexibility rather than global structural instability.

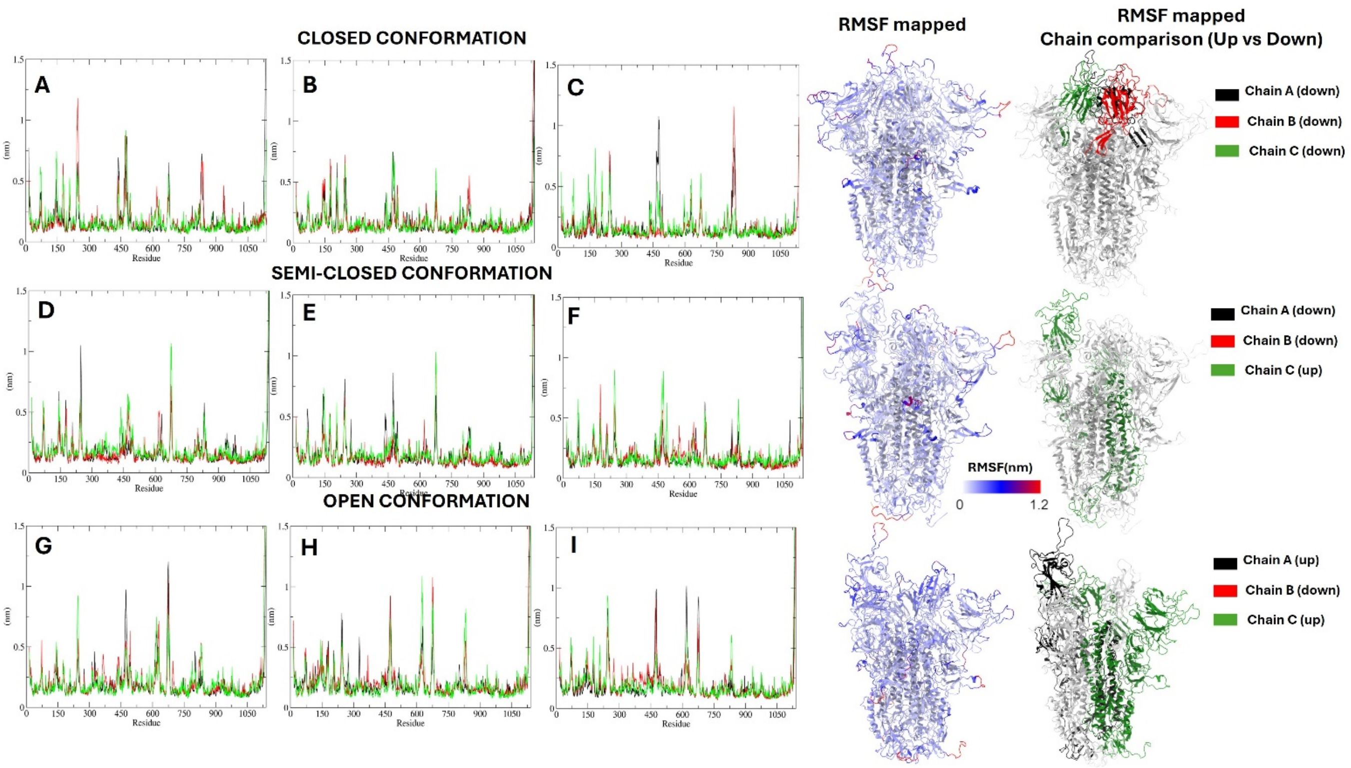

Figure S5. Residue-level flexibility analysis of the SARS-CoV-2 Spike protein across closed, semi-closed, and open conformations. (A–C) RMSF profiles for the closed conformation; (D–F) semi-closed conformation; (G–I) open conformation. Each panel represents an independent chain of the trimer (colored in black, red, and green). (Right panels) RMSF values mapped onto the three-dimensional structure of the Spike protein for each conformational state, illustrating the spatial distribution of residue flexibility. Warmer colors indicate higher fluctuations, while cooler colors represent more stable regions. Chain-specific representations highlight the relative positioning of protomers adopting "up" and "down" conformations, showing that residue fluctuations are primarily localized in surface-exposed regions, including loop regions, the N-terminal domain (NTD), and the receptor-binding domain (RBD).

Table S1.

Cluster size (%) of the dominant conformational states identified from molecular dynamics simulations of the SARS-CoV-2 JN.1 Spike protein. Clustering was performed using the GROMOS algorithm with an RMSD cutoff of 0.25 nm based on Cα atoms. Values represent the percentage of frames belonging to the most populated cluster for each independent replica (A–C) across the closed, semi-closed, and open conformations. Higher cluster populations indicate greater conformational stability and reduced structural dispersion during the simulation.

Conformation	Replica	Cluster size (%)
Closed	A	91
Closed	B	87.2
Closed	C	67.4
Semi-closed	A	95.4
Semi-closed	B	55.2
Semi-closed	C	92.8
Open	A	48.6
Open	B	44.2
Open	C	45

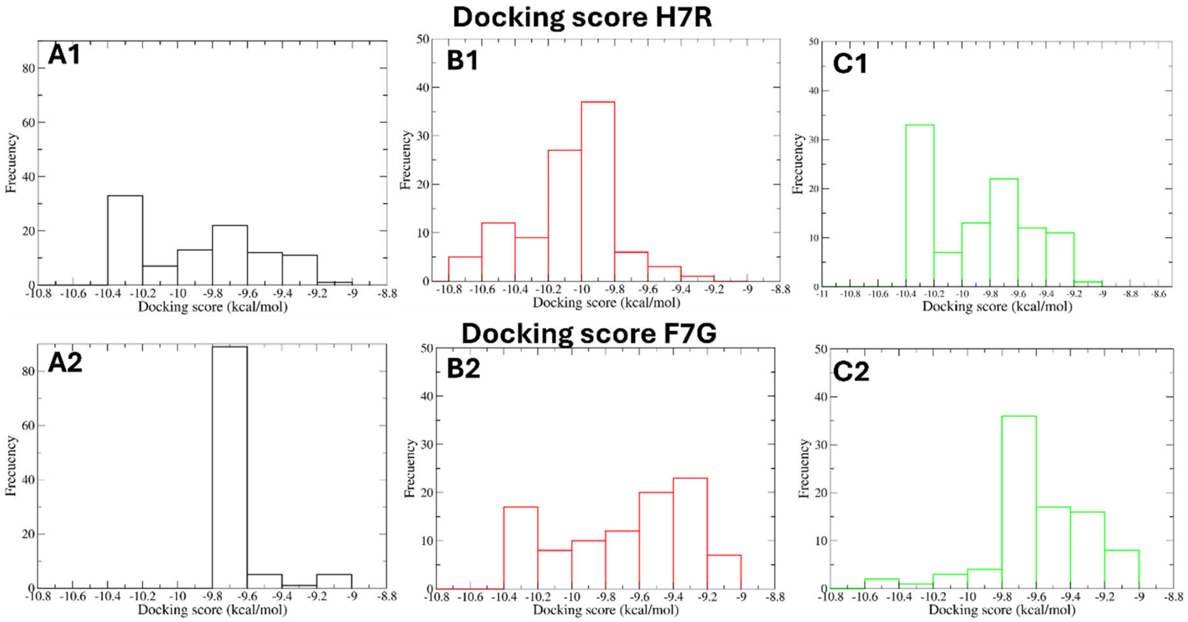

Figure S4. Distribution of docking scores obtained from 100 independent docking runs for H7R and F7G across closed, semi-closed, and open conformations of the SARS-CoV-2 JN.1 Spike protein. Histograms show the frequency of docking scores for each system, highlighting the concentration of poses within a narrow energy range across conformational states.

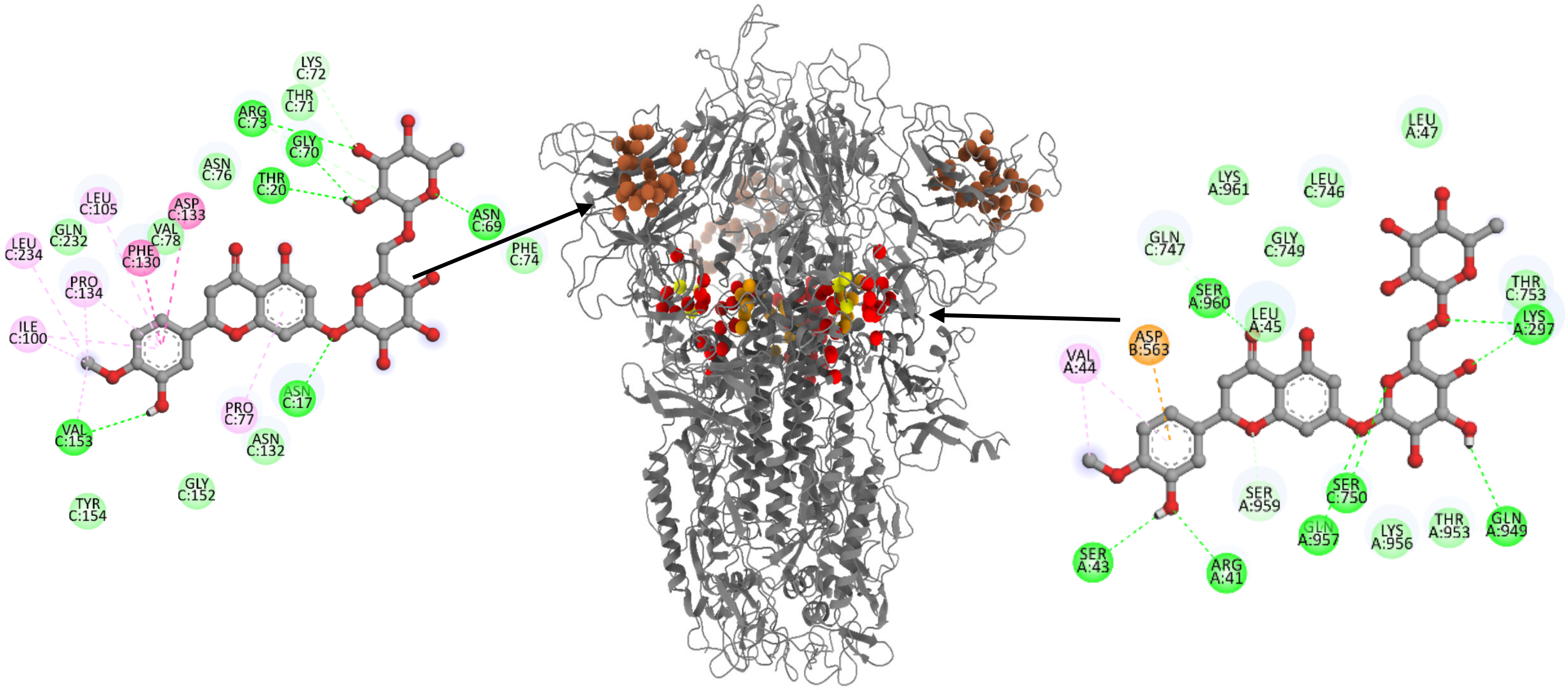

Figure S6. Structural clustering analysis of representative docking poses for H7R in the closed JN.1 Spike conformation. The most populated docking cluster (Cluster 1) is shown in red, yellow, and orange, representing recurrent ligand poses occupying the dominant interaction region across equivalent protomeric environments. The second most populated cluster (Cluster 2) is shown in brown. Representative two-dimensional interaction maps are provided for major clusters to illustrate differences in ligand orientation and residue contacts.

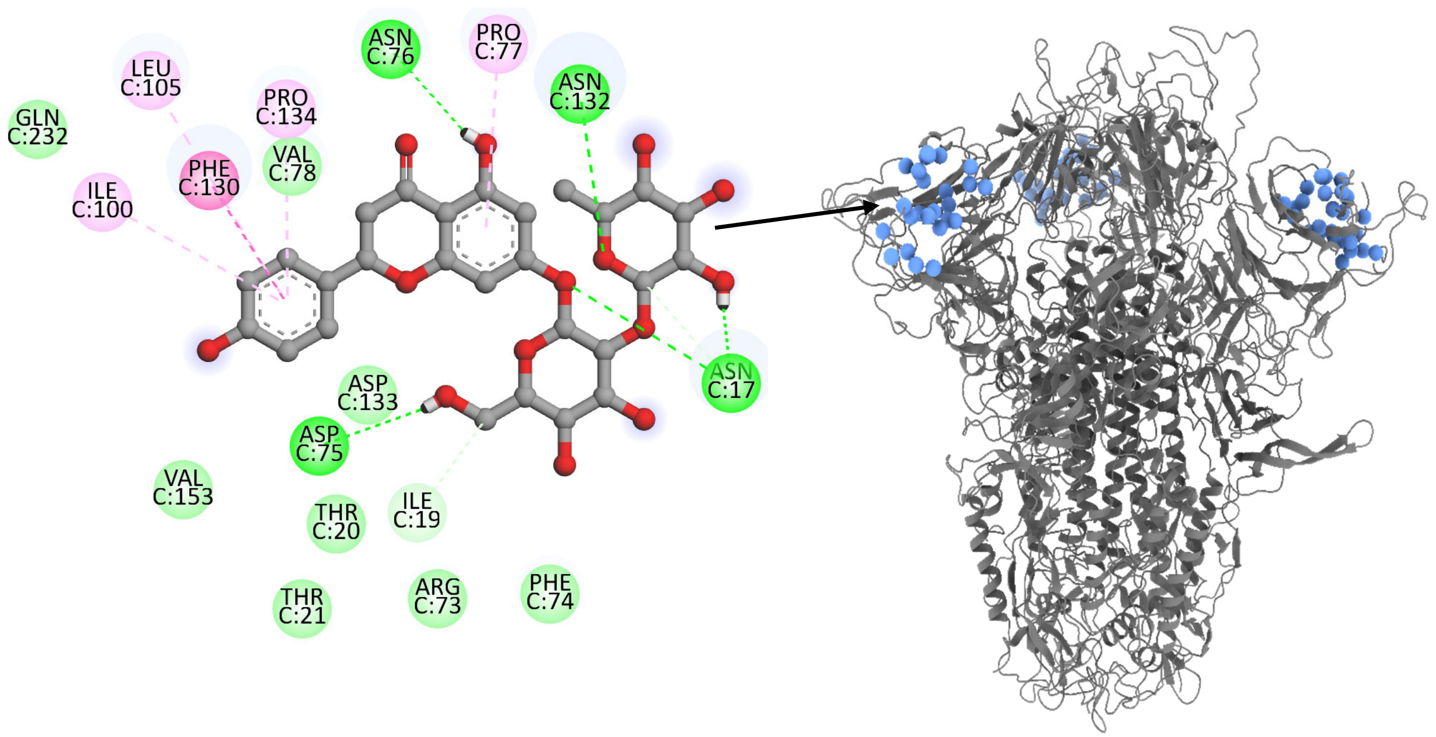

Figure S7. Dominant docking cluster for F7G in the closed conformation of JN.1 Spike protein. RMSD-based clustering analysis (2 Å cutoff) revealed a highly recurrent interaction mode representing 87% of all docking poses. The structural mapping (right) shows spatial convergence of representative poses within a defined ligand-accessible region of the S1 subunit across equivalent protomeric environments.

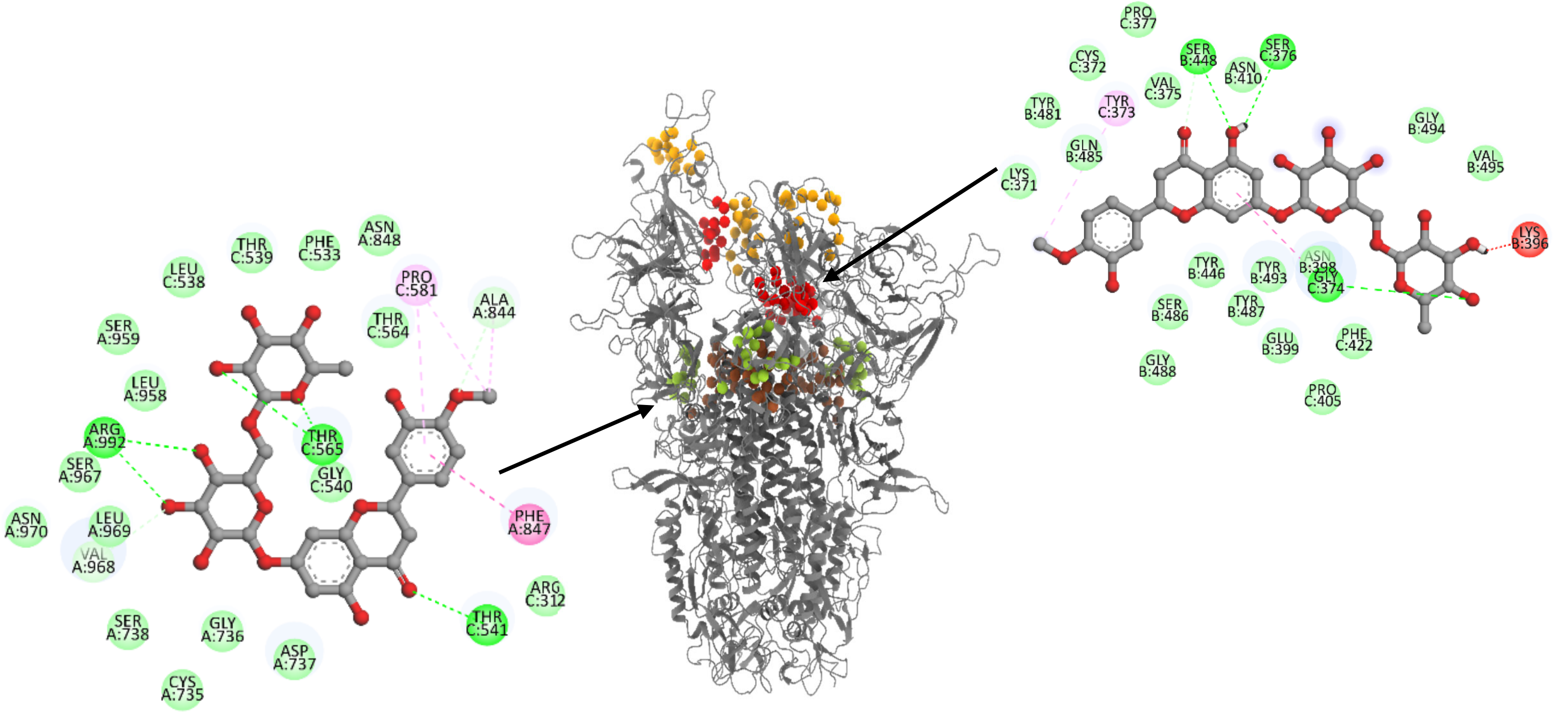

Figure S8. Structural clustering analysis of H7R docking poses in the semi-closed JN.1 Spike conformation. The most populated docking cluster (Cluster 1; red and orange) localizes predominantly in an exposed region of the receptor-binding domain (RBD), representing the dominant recurrent interaction mode identified by RMSD-based clustering (2 Å cutoff). The second most populated cluster (Cluster 2; brown and olive green) occupies a distinct central region within the S1 subunit, indicating an alternative but recurrent interaction mode. Representative two-dimensional interaction maps are shown for each major cluster, highlighting differences in ligand orientation and residue contact networks. These results demonstrate that while H7R preferentially associates with an exposed RBD-accessible region in the semi-closed conformation, secondary clusters can occupy structurally distinct regions, supporting the interpretation of docking clusters as recurrent spatial interaction modes rather than solely energy-dependent poses.

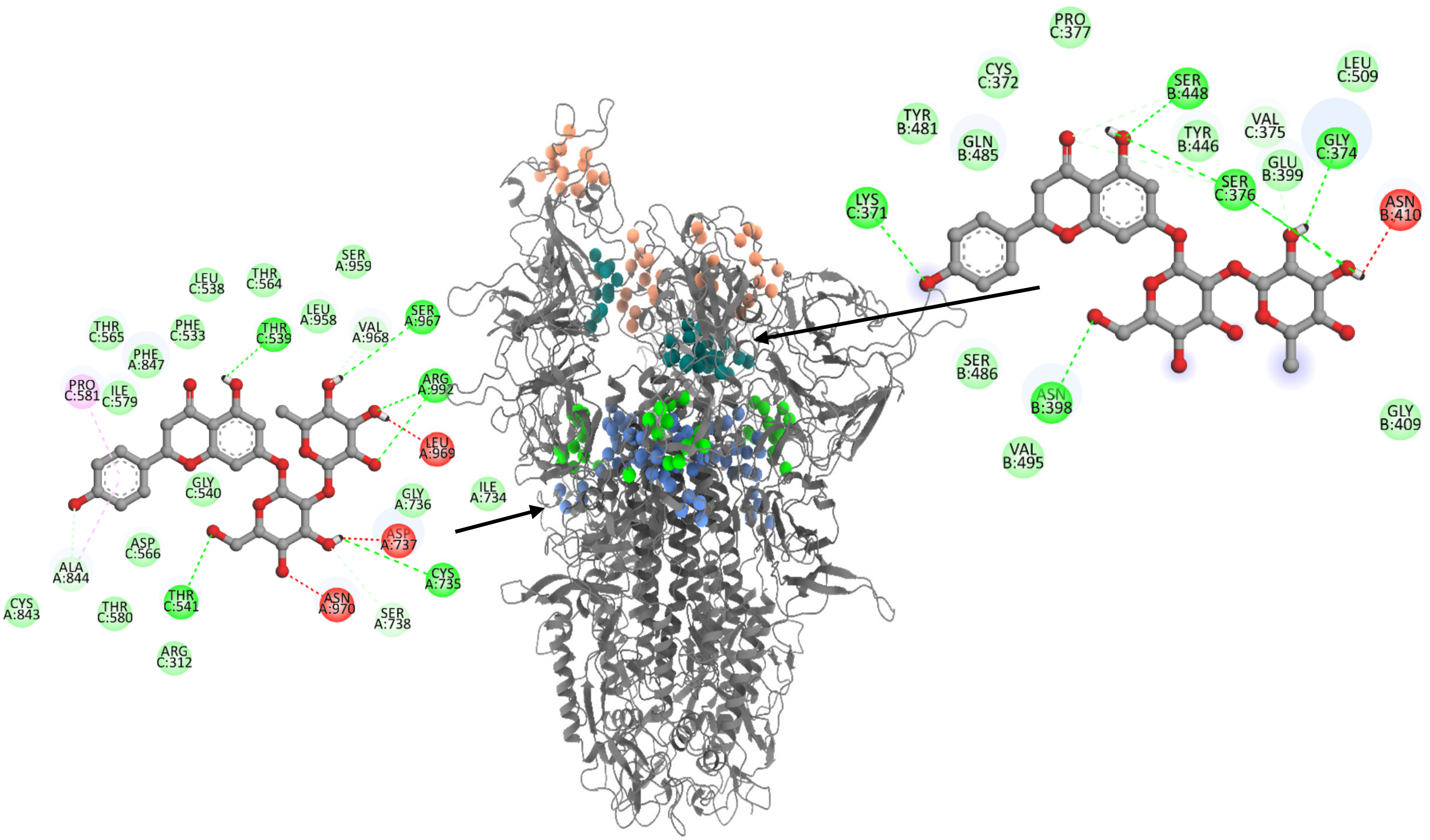

Figure S9. Structural clustering analysis of F7G docking poses in the semi-closed JN.1 Spike conformation. The most populated docking cluster (Cluster 1; lime green and light blue) occupies a recurrent central interaction region within the S1 subunit, representing the dominant spatial binding mode identified through RMSD-based clustering (2 Å cutoff). The second most populated cluster (Cluster 2; pink and dark blue) localizes predominantly in a more exposed region associated with the receptor-binding domain (RBD), indicating an alternative recurrent interaction mode. Representative two-dimensional interaction maps are shown for each major cluster, illustrating distinct ligand orientations and residue interaction networks within their respective binding environments. These results demonstrate that F7G in the semi-closed conformation samples multiple structurally defined ligand-accessible regions, with the dominant cluster favoring a central S1 interaction mode while secondary clusters retain accessibility to more exposed RBD-associated surfaces

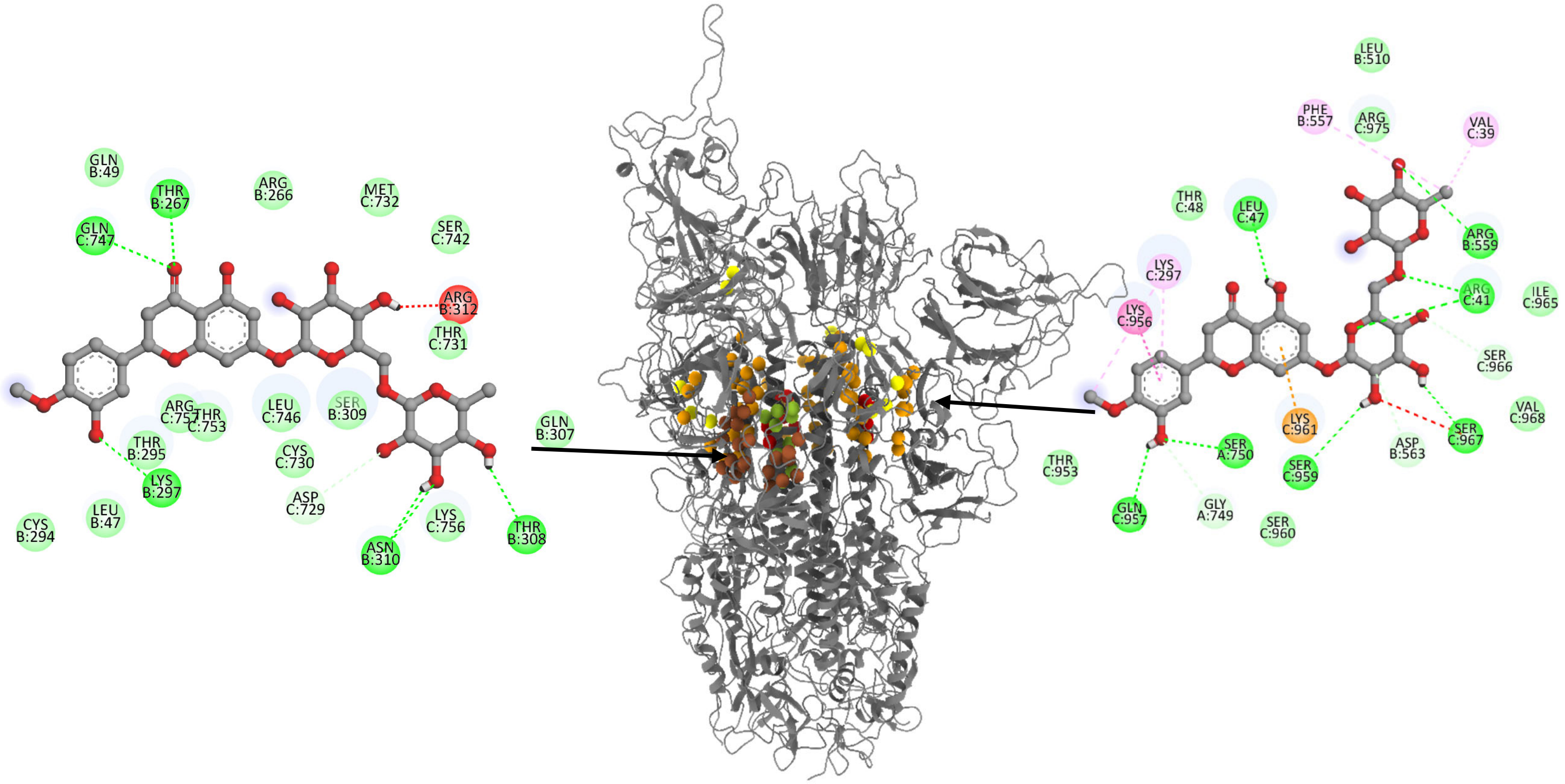

Figure S10. Structural clustering analysis of H7R docking poses in the open JN.1 Spike conformation. The most populated docking cluster (Cluster 1; yellow, orange, and red) occupies a dominant central interaction region within the S1 subunit, representing the principal recurrent interaction mode identified through RMSD-based clustering (2 Å cutoff). The second most populated cluster (Cluster 2; brown and olive green) localizes within a neighboring but partially overlapping region, indicating an alternative recurrent interaction mode within the same broader ligand-accessible environment. Representative two-dimensional interaction maps are shown for each major cluster, illustrating distinct ligand orientations and residue contact networks despite their spatial proximity. These results indicate that in the open conformation, H7R primarily samples a central ligand-accessible region while maintaining secondary orientation states within adjacent structural space, supporting the interpretation that dominant docking clusters may reflect multiple recurrent binding orientations within shared spatial environments rather than entirely independent binding sites.

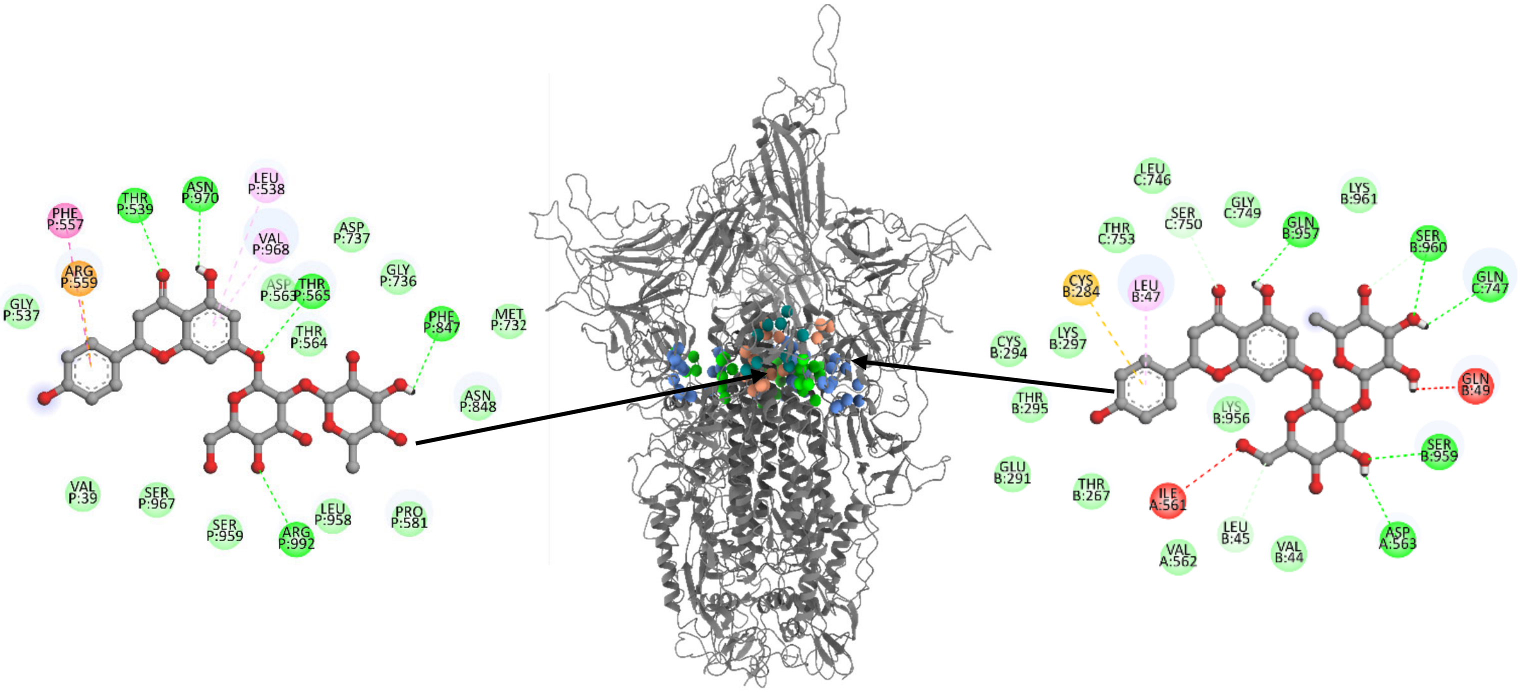

Figure S11. Structural clustering analysis of F7G docking poses in the open JN.1 Spike conformation. The most populated docking cluster (Cluster 1; light blue and lime green) defines the dominant recurrent interaction mode within a central ligand-accessible region of the S1 subunit, as identified through RMSD-based clustering (2 Å cutoff). The second most populated cluster (Cluster 2; pink and dark blue) is largely superimposed with Cluster 1, indicating that both clusters occupy the same primary spatial interaction environment while differing mainly in ligand orientation and local residue contact patterns. These results demonstrate that in the open conformation, F7G preferentially associates with a highly recurrent central binding region, where dominant and secondary clusters reflect alternative orientation states within the same spatially conserved docking environment rather than distinct independent binding sites.

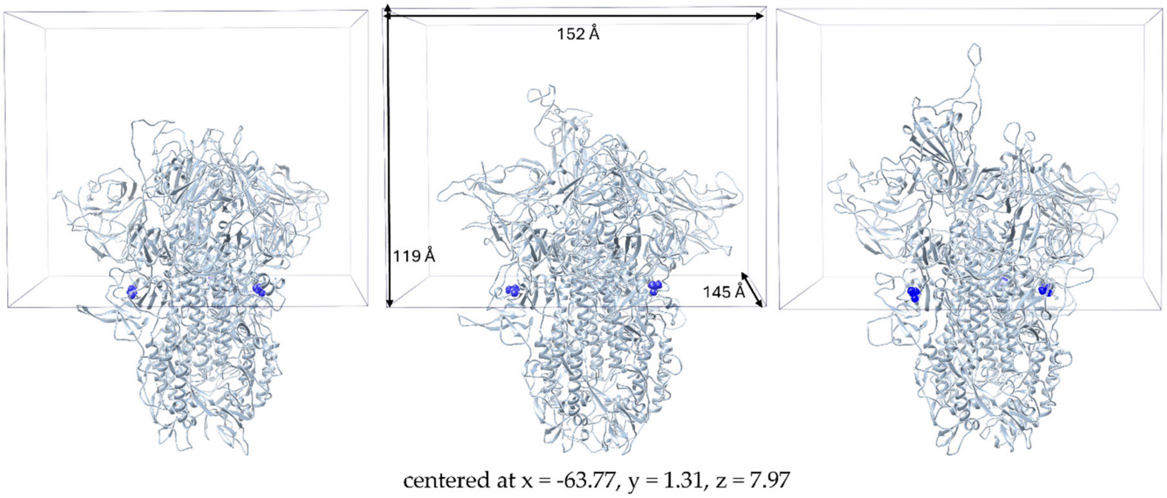

Figure S12. Structural definition of the docking search space used for blind docking analyses of the SARS-CoV-2 JN.1 Spike protein. Representative closed, semi-closed, and open Spike conformations are shown separately in the same structural orientation to illustrate the conformational breadth of the analyzed system. The docking search space (box) was designed to encompass the complete S1 subunit, including the N-terminal domain (NTD) and receptor-binding domain (RBD), ensuring broad and structurally consistent exploration of ligand-accessible regions across distinct conformational states. The dimensions of the docking box (152 × 119 × 145 Å) and its center coordinates (x = −63.77, y = 1.31, z = 7.97) are indicated. The search region was positioned using residue D614 as a structural reference point to maintain comparable spatial coverage across all conformations despite large-scale RBD rearrangements. This strategy allowed unbiased sampling of exposed S1 interaction landscapes rather than restricting docking to a predefined binding pocket.
